# Qing-Kai-Ling Injection Induces Immediate Hypersensitivity Reaction *via* the Activation of Anaphylatoxin C3

**DOI:** 10.3389/fphar.2019.01524

**Published:** 2020-01-09

**Authors:** Yuan Gao, Ruijuan Qi, Xiaoyu Zhang, Xudong Xu, Yixin Han, Qiaoling Fei, Xiaojing Wang, Runlan Cai, Guibo Sun, Yun Qi

**Affiliations:** ^1^ Institute of Medicinal Plant Development, Chinese Academy of Medical Sciences & Peking Union Medical College, Beijing, China; ^2^ Institute of Materia Medica, Chinese Academy of Medical Sciences & Peking Union Medical College, Beijing, China

**Keywords:** Qing-Kai-Ling injection, histamine, complement-derived anaphylatoxin C3, IgE, immediate hypersensitivity reactions

## Abstract

**Background and Objective:** Qing-Kai-Ling (QKL) is derived from a famous ancient Chinese patent medicine Angong Niuhuang pills (ANP) which has been used across Asia, especially in China, for the treatment of “febrile disease,” such as stroke, encephalitis and meningitis for hundreds of years. As an extract of ANP without heavy metal, the clinical applicability of QKL is more intensive, of which its injection is commonly used in acute and serious diseases. This study aims to clarify the potential mechanisms of immediate hypersensitivity reaction (IHR) induced by QKL injection (QKLI).

**Methods:** β-hexosaminidase release assay was performed on the human mast cell line LAD2 and mouse peritoneal mast cells. T helper 2 (Th2) immunity-amplified mice were prepared by aluminum adjuvant. Anaphylactic shock was detected by measuring rectal thermometry in propranolol-pretreated mice. For evaluating microvascular permeability, Evans Blue extravasation assay was used. Serum total IgE (tIgE) and the activated complement-derived anaphylatoxin C3 (C3a) levels were measured by ELISA.

**Results:** QKLI was unable to elevate serum tIgE level in the Th2 immunity-amplified mice, but can increase vasopermeability and trigger anaphylaxis after the first injection. By screening seven fractions of QKLI, only the extract of Isatidis Radix (*Isatis tinctoria L.*) induced hindpaw Evans Blue extravasation, which was disappeared in Isatidis Radix-free QKLI. Mechanism study indicated that QKLI or Isatidis Radix-caused IHR could be blocked by the antagonists for histamine or C3a, rather than PAF or C5a. Consistently, QKLI and Isatidis Radix could also directly activate human serum complement-derived anaphylatoxin 3 (C3) *in vitro* with the half effective concentration values of 0.69% and 218.6 μg/ml, respectively.

**Conclusion:** QKLI-IHR is complement activation-related pseudoallergy, rather than an IgE-mediated allergy. QKLI activates C3 and might consequently provoke mast cells to release histamine, which is a principal effector of its IHR. The pseudoallergic reaction induced by QKLI was attributed to the extract of Isatidis Radix. This study suggests a potential therapeutic strategy for the prophylaxis and treatment of QKLI-IHR.

## Introduction

Qing-Kai-Ling injection (QKLI), a notable antipyretic preparation, is derived from Angong Niuhuang pills (ANP). ANP is a famous ancient Chinese patent medicine for the treatment of “febrile disease,” and has been used across Asia, especially in China, for treating stroke, encephalitis and meningitis (Fu et al., 2017) for hundreds of years. For containing heavy metal (realgar and cinnabar), ANP is forbidden in the USA and European countries (Liu et al., 2008a; Liu et al., 2008b). As an extract of ANP without heavy metal (Topic Study Group in Beijing College of Traditional Chinese Medicine, 1975), Qing-Kai-Ling (QKL) is widely used for the treatment of the upper respiratory inflammation, pneumonia, viral encephalitis and high fever (Gao et al., 2018) and listed in the Chinese Pharmacopoeia (State Pharmacopoeia Committee, 2015). Its components include Isatidis Radix (*Isatis tinctoria L.*), Lonicerae japonicae Flos (*Lonicera japonica* Thunb.), Gardeniae Fructus (*Gardenia jasminoides* J.Ellis), powdered buffalo horn, Concha Margaritifera Usta, cholic acid, hyodeoxycholic acid and baicalin. Perhaps the change in the traditional drug-delivery way (p.o.) brings increasing rapidly cases of adverse drug reaction (ADR) (Li et al., 2010), and an alarm over QKLI-caused potential risks in patients was released by the Chinese National Center for Adverse Drug Reaction Monitoring in June 2008 and April 2009, respectively (National Center for ADR Monitoring, 2008; National Center for ADR Monitoring, 2009). In fact, QKLI is the second leading cause of ADRs induced by traditional Chinese medicine injections (Zhang et al., 2016).

Immediate hypersensitivity reaction (IHR) can be divided into allergic- and non-allergic (NA)-mediated (Johansson et al., 2001), while “anaphylaxis” is reserved for severe IHR (Johansson et al., 2004). Drugs are the most common anaphylaxis triggers in adults (Aun et al., 2017). Among QKLI-induced ADRs, IHR accounted for the largest proportion (Zhong et al., 2012). Accordingly, many researchers have focused on QKLI-induced IHR (QKLI-IHR). Some researchers speculated that QKLI caused allergic-IHRs based on the increased total IgE (tIgE) level in the serum of the QKLI-caused anaphylaxis patients (Zhao et al., 2011), while others thought that QKLI-IHR was a non-immune mediated reaction by virtue of the unchanged plasma tIgE level in Beagle dogs treated with QKLI (Wang et al., 2010). Seemingly, QKLI can lead to the degranulation of effector cells (e.g., RBL-2H3, etc.) directly (Chen et al., 2011; Cui et al., 2014). But basic secretagogues [e.g., compound 48/80 (C48/80) and substance P, etc.] activate human mast cells degranulation through a single membrane receptor MrgprX2, whose orthologue in murine is MrgprB2 (Grimbaldeston, 2015; McNeil et al., 2015; Ali, 2016). As a rat basophilic leukemia cell line, RBL-2H3 cannot respond to C48/80 owing to the lack of these endogenous receptors (Kashem et al., 2011; Subramanian et al., 2013), which is in agreement with our observational results. Thus, the attribution of QKLI-IHR and its underlying mechanisms are far from clear. The present study indicates that QKLI can directly activate complement-derived anaphylatoxin 3 (C3), which might subsequently stimulate its effector cells (e.g., mast cells and basophils) (Ali, 2010), thus releasing histamine to cause IHRs.

## Materials and Methods

### Materials and Reagents

Commercial QKLI (Batch nos. 16020204, 16020205, and 16020206) and its eight raw materials were provided by Yisheng Pharmaceutical Co., Ltd. (Ji’an, Jilin, China) and authenticated by the Quality Controller Guilan Ding according to the Chinese Pharmacopoeia (State Pharmacopoeia Committee, 2015). Four intermediate fractions in QKLI (F1, the extract of powdered buffalo horn and margaritifera concha; F2, the extract of Gardeniae Fructus; F3, the extract of Isatidis Radix; F4, the extract of Lonicerae japonicae Flos) were prepared according to the Chinese Pharmacopoeia (State Pharmacopoeia Committee, 2015). The used QKLI in this study is the mixture of three batches products. C48/80, 4-Methylumbelliferyl N-acetyl-β-D-glucosaminide, propranolol, triprolidine, CV3988, and SB290157 were purchased from Sigma-Aldrich (St Louis, MO, USA). PMX53 was from TOCRIS Bioscience (Bristol, UK). Rehydragel^®^ aluminum adjuvant was from General Chemical (Parsippany, NJ, USA). Mouse total IgE (tIgE) ELISA kit was from Biolegend Co. (San Diego, CA, USA). Human C3a ELISA kit was from BD Biosciences (San Diego, CA, USA). Shrimp tropomyosin (ST) from *Metapenaeus ensis* was prepared as we previously described (Gao et al., 2017). Normal human serum was obtained from Solarbio life sciences Co. (Beijing, China).

### HPLC Analysis

Commercial QKLI was assayed by a Waters HPLC system. A highresolution HPLC column (Tnature C18, 250 mm × 4.6 mm, 5 μm) were used. Mobile phase A was 0.1% v/v formic acid in water and mobile phase B was acetonitrile. The flow rate is 1 ml/min. A gradient of mobile phase A is 0–5 min (0%–2%), 5–8 min (2%–10%), 8–50 min (10%–30%) and 50–60 min (30%–100%). The UV spectrophotometer detector was set at 235 nm.

### Ethics Statement

All the animal experiments were carried out according to the National Institutes of Health Guide for the Care and Use of Laboratory Animals and approved by the Institutional Care and Use Committee, Institute of Medicinal Plant Development of Chinese Academy of Medical Sciences. Anesthetic drugs and all other necessary measures were used to reduce animal suffering during experimental procedures.

### Cells and Animals

The human mast cell line LAD2 (from Michael D. Gershon, MD, Columbia University, USA) was a gift from Prof. Renshan Sun (the Third Military Medical University, Chongqing, China). Balb/c mice (female, 18–20 g) were purchased from Vital River Experimental Animal Services (Beijing, China) and housed in an SPF laboratory under standard temperature (22–24°C) and humidity (45%–65%) conditions with a 12 h light/dark cycle and standard pallet diet and water ad libitum. Primary abdominal mast cells were obtained from Balb/c mice.

### Measurement of Serum tIgE

Balb/c mice were injected (i.p.) weekly with aluminum adjuvant (100 μl/mouse) containing QKLI (50 μl/mouse) or ST (60 μg/mouse). ST was used as a positive control allergen. The negative control mice were only treated with aluminum adjuvant. Five weeks later, the mice were euthanized and the serum was collected for the assay. Serum tIgE level was determined by using a commercial ELISA kit according to the assay protocol.

### β-Hexosaminidase Release Assay

To determine the direct effect of QKLI on cell degranulation, LAD2 cells (5 × 10^4^ cells/well) or mouse peritoneal mast cells (1 × 10^6^ cells/well) were seeded in the 96-well plates and treated with QKLI at 37°C for 1.5 h. To evaluate the inhibitory effect of QKLI on C48/80-induced degranulation, the cells were pretreated with QKLI for 30 min followed by adding C48/80 (10 μg/ml) for further 1.5 h incubation. 30 μl of the supernatant was transferred to a 96-well black flat-bottom plate accompany with 50 μl of substrate solution (0.57 mg/ml 4-Methylumbelliferyl N-acetyl-β-D-glucosaminide in the buffer contained 133 mM sodium citrate and 133 mM NaCl, pH 4.3). The reaction proceeded at 37°C for 1.5 h and was stopped by adding stop buffer (50 mM glycine and 5 mM EDTA-Na2, pH 10.5; 200 μl/well). Fluorescence was determined with a fluorescence microplate reader at λex 355 nm and λem 460 nm (Gao et al., 2017).

### Evans Blue Extravasation Assay

Evans Blue extravasation in mice hind paws was measured as previously described (McNeil et al., 2015). Briefly, 15 min after induction of anesthesia (50 mg/kg of pentobarbital), mice were intraplantarly (left paw) injected with 7 μl of QKLI. The right paw was injected with 7 μl of NS. Five minutes later, the mice were injected (i.v.) with 100 μl of 6.25 mg/ml Evans Blue. Thirty minutes later, the mice were euthanized. The paw tissues were collected and weighed. Evans Blue in the paw tissues were extracted by formamide at 60°C for 24 h. The OD values were read at 620 nm. The concentration of the dye in the paw tissues was calculated by the standard curve of the Evans Blue dye, and the dye content was expressed in microgram per gram of tissue.

### Anaphylactic Shock Assay

Anaphylactic shock was assessed by rectal thermometry (Strait et al., 2002). To increase the severity of anaphylaxis (TenBrook et al., 2004; Khodoun et al., 2009), the mice were pretreated (i.v.) with propranolol (35 μg/mouse). Twenty minutes later, the mice were challenged (i.p.) with normal saline (NS) or QKLI (100 μl/mouse). Thirty minutes later, the rectal temperature was measured. For the antagonist experiment, CV3988 (a PAF antagonist, 0.337 μmol/mouse) or triprolidine (an H1 antagonist, 0.64 μmol/mouse) was injected (i.p.) into the mice 10 min before the propranolol pretreatment.

### Complement Activation Assay *In Vitro*

The human serum (100 μl, 1:500 dilution) was mixed with 4 μl of CaCl_2_ (1 M), 4 μl of MgCl_2_ (1 M), and 10 μl of test substances, and then incubated at 37°C. After 20 min, the reaction was stopped using 22 μl of EDTA (0.5 M, pH 8.0) and cooling to 0°C. Tween-80 was used as a positive control (Weiszhár et al., 2012). The level of C3a in the plasma was determined using a commercial ELISA kit according to the manufacturer’s instructions.

### Statistical Analysis

Data are expressed as the means ± SD from at least three independent experiments and were analyzed by a one-way analysis of variance (ANOVA). A Student *t*-test was used when only two groups were compared. A difference with a *P*-value < 0.05 was considered statistically significant.

## Results

### Fingerprint of QKLI

**Figure 1:**
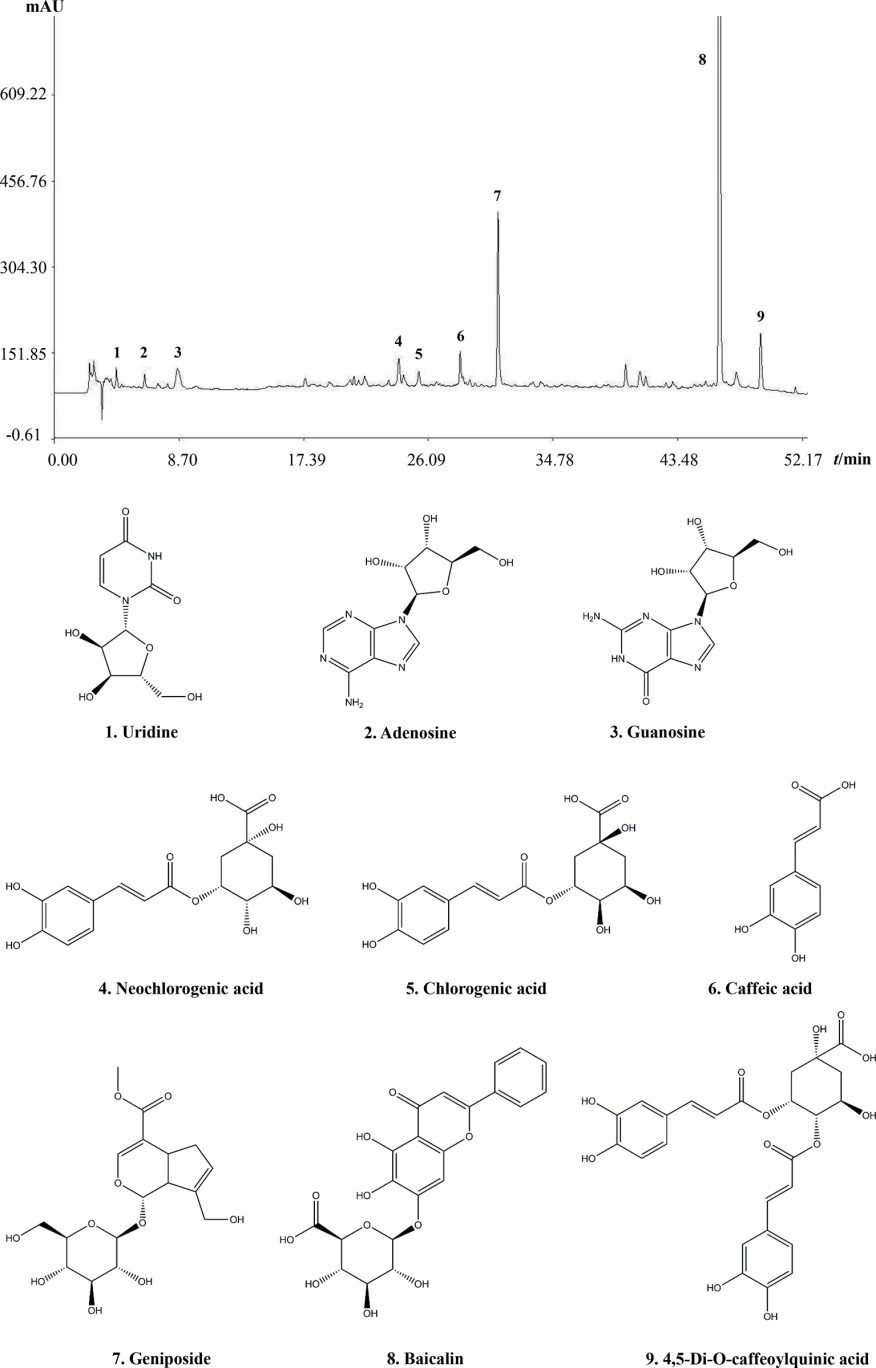
Chromatogram fingerprint of Qing-Kai-Ling Injection.

The main component profiles of QKLI were analyzed via HPLC-UV. The retention time values of the identified compounds were compared with that of the reference substances. Nine components (uridine, adenosine, guanosine, neochlorogenic acid, chlorogenic acid, caffeic acid, geniposide, baicalin, and 4,5-Di-O-caffeoylquinic acid) in QKLI were identified and their structures had also been shown (**Figure 1**).

### QKLI Fails to Induce IgE-Mediated IHR but Can Cause Non-Allergic IHR

**Figure 2:**
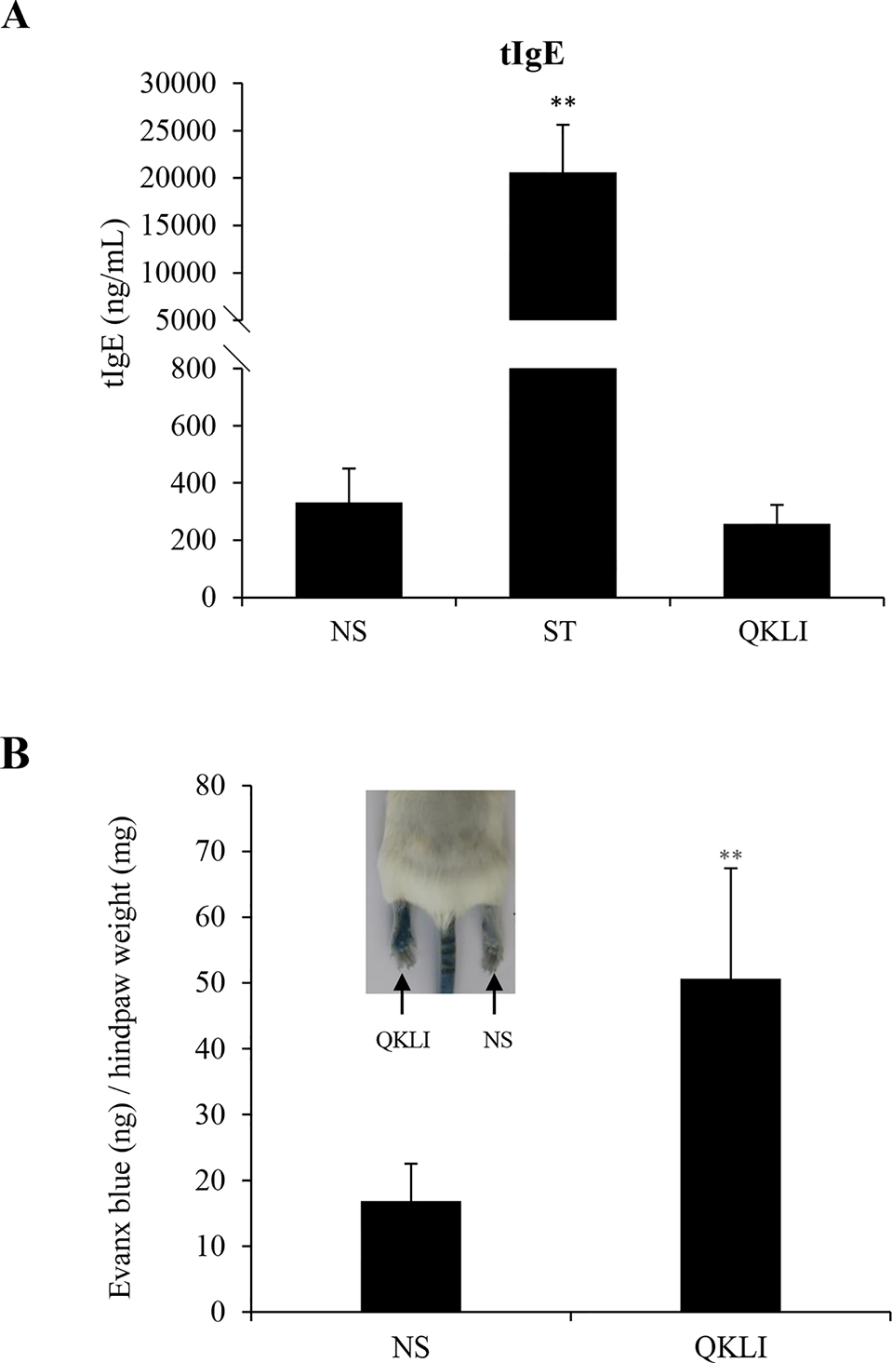
QKLI fails to induce IgE-mediated IHRs but can cause NA-IHRs. **(A)** QKLI failed to elevate mouse serum tIgE. Mice were injected weekly (i.p.) with aluminum adjuvant (100 μl/mouse) containing QKLI (50 μl/mouse) or ST (60 μg/mouse). Five weeks later, the serum tIgE level was determined using a commercial ELISA kit. ^**^ *P* < 0.01 *vs.* NS. **(B)** QKLI induced vasopermeability increase after the first intraplantar injection. Fifteen minutes after induction of anesthesia (50 mg/kg of pentobarbital), mice were intraplantarly (left paw) injected with 7 μl of QKLI. The right paw was injected with 7 μl of NS. Five minutes later, the mice were injected (i.v.) with 100 μl of 6.25 mg/ml Evans Blue. Thirty minutes later, the mice were euthanized. The paw tissues were collected and weighed. Evans Blue in the paw tissues were extracted by formamide at 60°C for 24 h. The OD values were read at 620 nm. The concentration of the dye in the paw tissues was calculated by the standard curve of the Evans Blue dye, and the dye content was expressed in microgram per gram of tissue. ^**^ *P* < 0.01 *vs.* NS. QKLI, Qing-Kai-Ling Injection; IHR, immediate hypersensitivity reaction; NA-IHR, non-allergic IHR; tIgE, total IgE; NS, normal saline.

IgE-mediated IHRs are the most common allergic-IHRs (Strait et al., 2006). To amplify the possible T helper 2 (Th2) response, the aluminum adjuvant was used during mouse immunization. Five weeks after intraperitoneal immunization, serum tIgE level was robustly elevated by ST, while QKLI failed to increase serum tIgE (**Figure 2A**), indicating that QKLI-IHR is not mediated by IgE. Next, we evaluated whether QKLI could induce non-allergic IHR (NA-IHR). The local effect of QKLI on microvascular permeability was determined using the Evans Blue extravasation assay. As shown in **Figure 2B**, QKLI significantly induced vasopermeability increase after the first intraplantar injection, demonstrating that QKLI-IHR was non-immune-mediated.

### Histamine H1 Receptor Antagonist Counters QKLI Induced NA-Anaphylaxis

**Figure 3:**
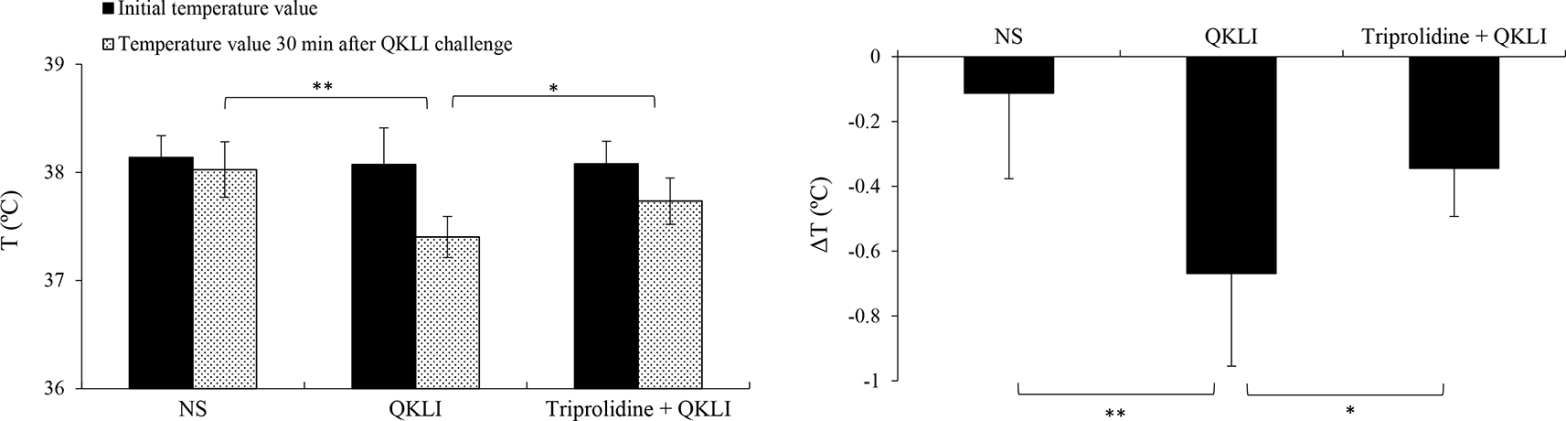
QKLI-induced hypothermia can be countered by triprolidine in propranolol-pretreated mice (n = 8). Triprolidine (0.64 μmol/mouse) or NS was injected (i.p.) into the mice. Ten minutes later, the mice were pretreated (i.v.) with propranolol (35 μg/mouse). Twenty minutes later, the mice were injected (i.p.) with NS or QKLI (100 μl/mouse). The rectal temperature was measured 30 min later. ^*^ *P* < 0.05 and ^**^ *P* < 0.01. QKLI, Qing-Kai-Ling Injection; NS, normal saline.

Considering that QKLI was capable of causing anaphylaxis clinically (National Center for ADR Monitoring, 2008; National Center for ADR Monitoring, 2009), the effect of QKLI on mouse rectal temperature was evaluated after a single injection (i.p.). Propranolol, which does not induce shock by itself, was used for increasing the severity of QKLI-induced anaphylaxis (TenBrook et al., 2004; Khodoun et al., 2009). As shown in **Figure 3**, C48/80 and QKLI contributed to obvious hypothermia in propranolol-pretreated mice.

Mast cells, basophils, and macrophages contribute predominantly to the pathogenesis of anaphylaxis through their secretion of histamine or/and PAF (Strait et al., 2002; Tsujimura et al., 2008). Thus, we used a PAF antagonist CV3988 and a histamine H1 receptor antagonist triprolidine for blocking QKLI-caused hypothermia. As a result, QKLI-induced anaphylaxis was not affected by CV3988 (data not shown), but significantly countered by triprolidine (**Figure 3**), indicating that histamine, rather than PAF, was the principal effector in QKLI-caused NA-anaphylaxis.

### QKLI Triggers NA-IHR by Activating Anaphylatoxin C3

**Figure 4:**
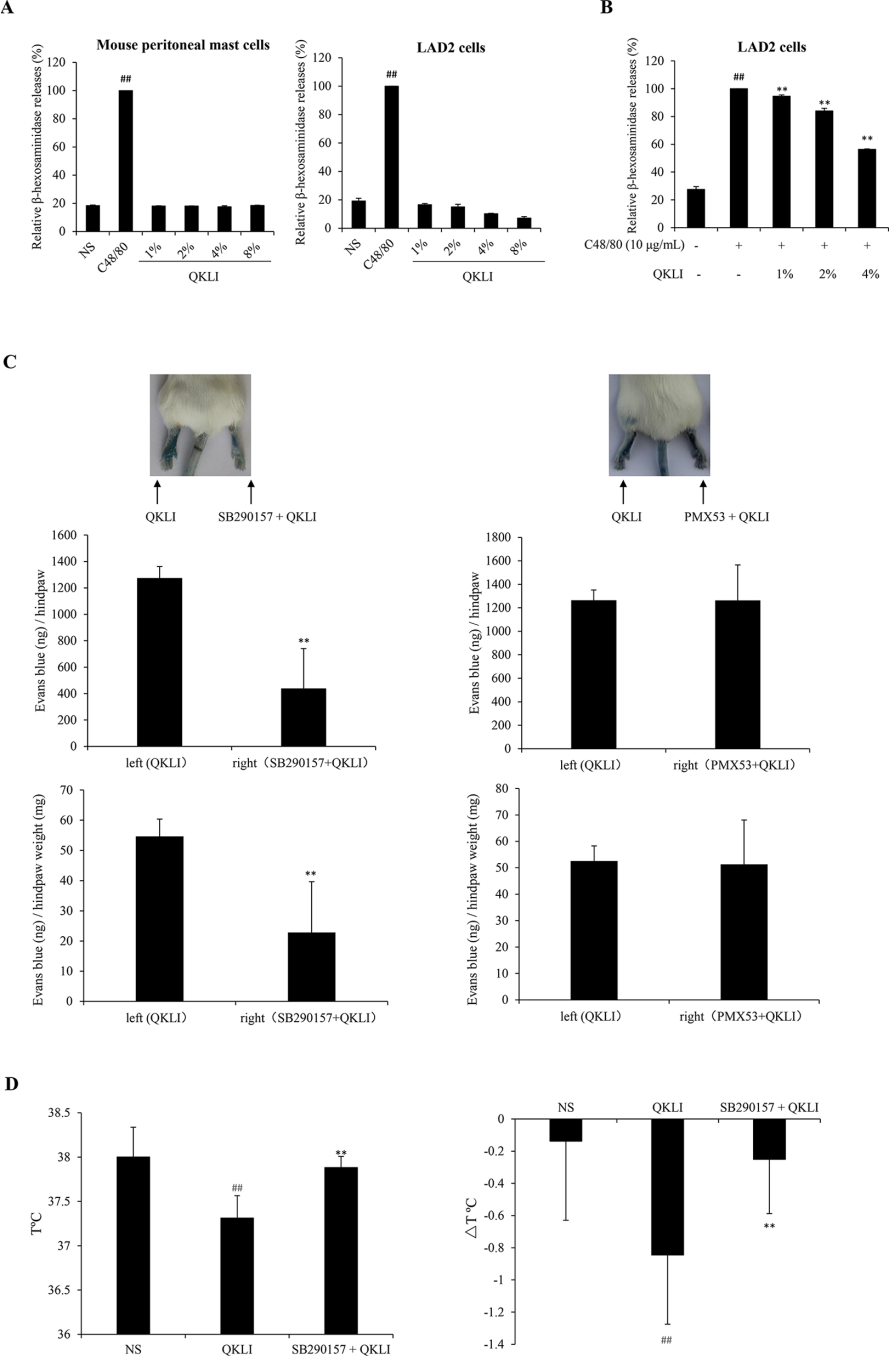
QKLI-induced NA-IHRs is mediated by C3a. **(A)** Effect of QKLI on the β-hexosaminidase release in mouse peritoneal mast cells and LAD2 cells. Cells were treated with QKLI at the indicated concentrations. Supernatant β-hexosaminidase level was determined 1.5 h later. C48/80 (10 μg/ml) was used as a positive control. ^##^ *P* < 0.01 *vs.* NS group. **(B)** QKLI decreased C48/80-induced β-hexosaminidase release in LAD2 cells. Cells were pretreated with QKLI for 30 min followed by adding C48/80 (10 μg/ml). Supernatant β-hexosaminidase level was determined 1.5 h later. ^##^ *P* < 0.01 *vs.* NS group and ^**^ *P* < 0.01 *vs.* C48/80 alone. **(C)** QKLI-induced Evans Blue extravasation was attenuated by SB290157 but not PMX53 (n = 5). Fifteen minutes after induction of anesthesia (50 mg/kg of pentobarbital), mice were intraplantarly injected with 15 μl of SB290157 (2 mg/ml) or PMX53 (1 mg/ml) or NS. Fifteen minutes later, the mice were intravenously injected with 100 μl of 6.25 mg/ml Evans Blue. Five minutes later, 7 μl of QKLI was administered by intraplantar injection in the hindpaws. Thirty minutes later, the mice were euthanized. The paw tissues were collected and weighed. Evans Blue was extracted by formamide at 60°C for 24 h. The OD values were read at 620 nm. The concentration of the dye in the paw tissues was calculated by the standard curve of the Evans Blue dye, and the dye content was expressed in microgram per gram of tissue. **P < 0.01 *vs.* QKLI alone. **(D)** SB290157 could block QKLI-induced anaphylaxis (n = 8). The mice were pretreated with propranolol (intravenously, i.v., 35 μg/mouse) 10 min after intraperitoneally injected with SB290157 (a C3a antagonist, 30 mg/kg). Twenty minutes later, the mice were intraperitoneally challenged with QKLI (100 μl/mouse). Thirty minutes later, the rectal temperature was measured. ^##^ *P* < 0.01 *vs.* NS and **P < 0.01 *vs.* QKLI alone. QKLI, Qing-Kai-Ling Injection; NA-IHR, non-allergic IHR; NS, normal saline.

Given that histamine is mainly released by the activated mast cells (Borriello et al., 2017), we firstly determined whether QKLI could directly promote mast cell degranulation. As shown in **Figure 4A**, 10 μg/ml of C48/80 evoked a markedly β-hexosaminidase release in both human LAD2 cells and mouse peritoneal mast cells, while QKLI could not induce mast cell degranulation. And not only that, it can dampen C48/80-induced degranulation concentration-dependently in LAD2 cells (**Figure 4B**) without cytotoxicity (data not shown), showing a potent mast cell stabilization effect.

To our knowledge, anaphylatoxin C3a or C5a can activate mast cells to secrete histamine (Johnson et al., 1975). Perhaps the QKLI-IHR is mediated by the activated complement. Accordingly, we used a C3a antagonist (SB290157) and a C5a antagonist (PMX53) to antagonize QKLI-induced vascular permeability increase. The result showed that SB290157, but not PMX53, significantly attenuated QKLI-induced Evans Blue extravasation (**Figure 4C**). Consistently, in an anaphylactic shock assay, QKLI-induced anaphylaxis could also be countered by SB290157 (**Figure 4D**), showing QKLI-caused NA-IHR was attributed to C3a, rather than C5a.

### Isatidis Radix Is Responsible for QKLI-IHR

**Figure 5:**
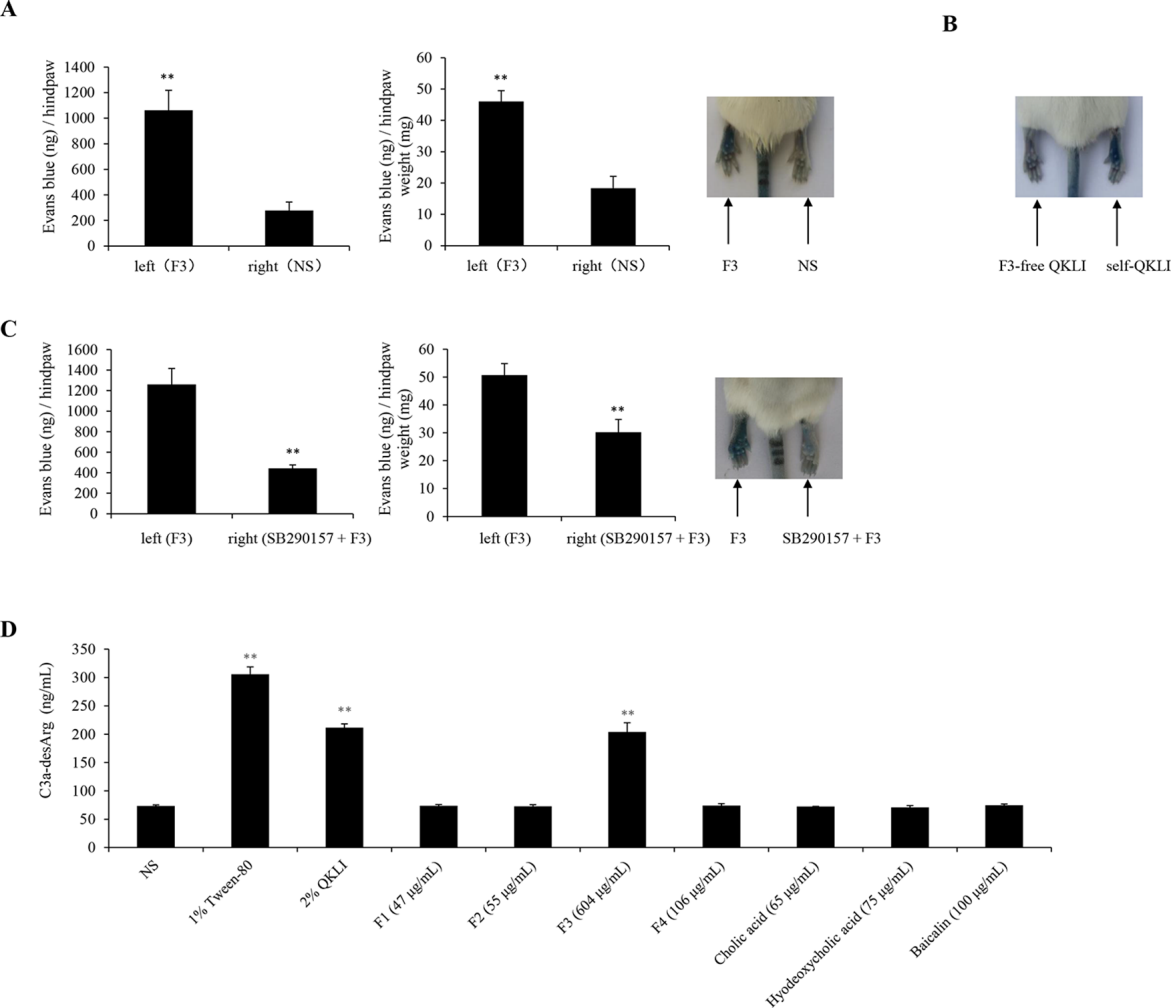
Isatidis Radix is responsible for QKLI-IHR. **(A)** F3 increased vasopermeability after the first intraplantar injection (n = 5). Fifteen minutes after induction of anesthesia (50 mg/kg of pentobarbital), mice were intraplantarly (left paw) injected with 7 μl of F3 (30.2 mg/ml). The right paws were injected with 7 μl of NS. Five minutes later, the mice were injected (i.v.) with 100 μl of 6.25 mg/ml Evans Blue. Thirty minutes later, the mice were euthanized. The paw tissues were collected and weighed. Evans Blue was extracted by formamide at 60°C for 24 h. The OD values were read at 620 nm. The concentration of the dye in the paw tissues was calculated by the standard curve of the Evans Blue dye, and the dye content was expressed in microgram per gram of tissue. ^**^ *P* < 0.01 *vs.* NS. **(B)** Representative image of Evans Blue extravasation of mouse paw induced by F3-free QKLI and self-QKLI. **(C)** F3-induced Evans Blue extravasation was attenuated by SB290157 (n = 5). ^**^ *P* < 0.01 *vs.* F3 alone. **(D)** QKLI and F3 directly activated C3 *in vitro*. The plasma was treated with test substances at the indicated concentrations and C3a-desArg level was determined by a commercial ELISA kit. Tween-80 was used as a positive control. ^**^ *P* < 0.01 *vs.* NS.

To identify the main constituent(s) causing QKLI-IHR, all intermediate fractions in QKLI, including four extracts (F1–F4) and three compounds (cholic acid, hyodeoxycholic acid, and baicalin), were ready according to the Chinese Pharmacopoeia (State Pharmacopoeia Committee, 2015), and respectively evaluated their potential ability to cause IHRs. As a result, at their equivalent concentrations in QKLI, only F3 (Isatidis Radix) markedly caused Evans Blue leakage into the paw after the first intraplantar injection (**Figure 5A**), and other fractions could not increase vasopermeability (data not shown). To confirm the above finding, QKLI without F3 (F3-free QKLI) and QKLI (self-QKLI) were prepared (235 mg F1, 275 mg F2, 3.02 g F3, 530 mg F4, 325 mg cholic acid, 375 mg hyodeoxycholic acid, and 500 mg baicalin were dissolved in water for injection to make 100 ml, pH 7.4). In agreement with commercial QKLI, self-QKLI, rather than F3-free QKLI, also potently induced hindpaw Evans Blue extravasation (**Figure 5B**). These results indicated that Isatidis Radix was the crime culprit of QKLI-IHR. This finding, together with complement activation mechanism of QKLI-IHR, urged us to use a C3a antagonist SB290157 for the next experiment. Not surprisingly, SB290157 significantly weakened F3-caused Evans Blue leakage into the paw (**Figure 5C**). *In vitro* results also showed that QKLI and F3 directly activated C3 in a concentration-dependent manner (**Figure 5D**) with the half effective concentration (EC_50_) values of 0.69% and 218.6 μg/ml, respectively.

To search for the definite ingredients for QKLI-IHR, we also tested nine availably characteristic compounds of Isatidis Radix, including indirubin, epigoitrin, DL-goitrin, 2-(1H-indol-3-yl)acetonitrile, indigo, chlorogenic acid, (1-methoxy-1H-indol-3-yl) acetonitrile, caffeic acid, and geniposide. Regrettably, none of them induced vasopermeability increase at a dose of 10 mM which is far higher than their equivalent concentrations in QKLI (data not shown), indicating that the 9 compounds are not the material basis of QKLI-IHR.

## Discussion

IHRs can be divided into allergic- and as non-allergic-mediated (Johansson et al., 2001). Most of QKLI-IHRs are likely to be non-allergic owing to the fact that QKLI-IHRs commonly occurred after the first treatment clinically (Xiang, 2010), but allergic-mediated IHRs cannot be ruled out yet. There are two pathways that can lead to allergic-mediated IHRs: the IgE-mediated classic pathway and the IgG-mediated alternative pathway (Strait et al., 2002). Although the kinetics and clinical features of these two types are generally similar, IgE is easier to cause allergy because considerably more antibody and antigen are required to induce IgG-mediated allergy (Strait et al., 2006). Thus, the potentiality of IgE-mediated allergy by QKLI was firstly evaluated. To amplify the possible Th2 response, an aluminum adjuvant was used during mouse immunization. Serum tIgE level was measured after 5 weeks, and during this period the tIgE concentration should be peak and stable (Gao et al., 2018). By contrast to ST, QKLI did not increase serum tIgE under our experimental conditions (**Figure 2A**), suggesting that it seems very unlikely to induce allergy by QKLI.

Consistent with clinical practice, QKLI can significantly contribute to NA-IHRs in both local (increase of microvascular permeability, **Figure 2B**) and systemic (induction of hypothermia, **Figure 3**) models. A histamine H1 receptor antagonist triprolidine can counter QKLI-induced NA-IHRs (**Figure 3**). Histamine is primarily secreted by mast cells (Borriello et al., 2017), suggesting that QKLI-IHRs might be mast cell-dependent. Mast cells can be activated by basic secretagogues (e.g., C48/80 or substance P) through a single receptor Mas-related gene receptor MrgprX2 (McNeil et al., 2015), which raises the question whether that QKLI possesses the characteristics of basic secretagogues. For the reason, two cells (human mast cell line LAD2 and mouse peritoneal mast cells) with the specific receptor MrgprX2 or MrgprB2 were used (Subramanian et al., 2013). As a result, QKLI could not induce two cells degranulation (**Figure 4A**). In C48/80-simulated LAD2 cells, QKLI even dampened β-hexosaminidase release (**Figure 4B**), indicating that QKLI induces NA-IHR via an indirect rather than direct effect on mast cells.

The complement-derived anaphylatoxins C3a and C5a can activate mast cells by binding to their respective receptors C3aR and C5aR and consequently lead to degranulation and histamine secretion (Ali, 2010). QKLI-IHR was countered by a C3a antagonist SB290157 (**Figures 4C**, **D**), rather than C5a antagonist (PMX53), demonstrating that QKLI might directly activate complement C3 in circulatory system. Indeed, QKLI directly activated C3 in human serum *in vitro* with an EC_50_ value of 0.69% (**Figure 5D**). According to the Chinese Pharmacopoeia (State Pharmacopoeia Committee, 2015), QKLI consists of seven fractions including four extracts and three compounds, of which F3 (the extract of Isatidis Radix), rather than other six fractions, potently induced Evans Blue extravasation in mouse hindpaw. This effect was disappeared in F3-free QKLI (**Figures 5A**, **B**) and also could be countered by the C3a antagonist (**Figure 5C**), indicating that the crime culprit for QKLI-IHR is Isatidis Radix, a non-ANP-derived material. Some components from Isatidis Radix, including baicalin and the other two botanical drugs (Gardeniae Fructus and Lonicerae japonicae Flos), were identified by HPLC (**Figure 1**). Next, we determined the effects of the available components from Isatidis Radix on the vascular permeability. Regrettably, the main ingredient(s) responsible for the QKLI-IHR was not found out. Accordingly, further chemical research is needed to obtain more new components of Isatidis Radix for identifying its pseudoallergic substances. In fact, QKLI-IHR is complement activation-related pseudoallergy (CARPA) (Szebeni, 2005), which was also commonly triggered by many chemical intravenous preparations, such as intravenous iron (Hempel et al., 2017), radiocontrast agents (Szebeni, 2001), etc. Obviously, as an extract of ANP, QKL induces an emerging CARPA which resulted from the addition of Isatidis Radix and the change of traditional drug-delivery way (p.o.). In the current situation, the pseudoallergic potency of Isatidis Radix should be emphatically monitored because it determines the intensity of QKLI-IHR.

In conclusion, this is the first study to show that QKLI-IHR is CARPA, rather than an IgE-mediated allergy. QKLI causes C3 activation, and might consequently activate mast cells to release histamine, which is a principal effector of its NA-IHR. In the seven fractions of QKLI, Isatidis Radix is primarily responsible for QKLI-IHR. Further explorations are required to identify the basis material associated with Isatidis Radix-induced NA-IHR. Our study suggests a potential therapeutic strategy for the prophylaxis and treatment of QKLI-IHR.

## Data Availability Statement

The raw data supporting the conclusions of this article will be made available by the authors, without undue reservation, to any qualified researcher.

## Author Contributions

YG and YQ wrote the manuscript. YG, RQ, XZ, XX, YH, QF, and XW performed the experiments. RC analyzed the raw data. GS and YQ designed the study and reviewed the manuscript. All authors read and approved the final version of the manuscript.

## Funding

This work was supported by the National S&T Major Project and Scientific Researchers Aiding Enterprise Item from the Ministry of Science and Technology of the People’s Republic of China (No. 2015ZX09501004-001-003), the CAMS Innovation Fund for Medical Sciences (2016-I2M-3-015), the National Natural Science Foundation of China (Nos. 81601385 and 81873066), and the Drug Innovation Major Project (No. 2018ZX09711001-009-012).

## Conflict of Interest

The authors declare that the research was conducted in the absence of any commercial or financial relationships that could be construed as a potential conflict of interest.
